# Facile Fabrication of Single-Walled Carbon Nanotube/Anatase Composite Thin Film on Quartz Glass Substrate for Translucent Conductive Photoelectrode

**DOI:** 10.3390/nano11123352

**Published:** 2021-12-10

**Authors:** Yutaka Suwazono, Takuro Murayoshi, Hiroki Nagai, Mitsunobu Sato

**Affiliations:** 1 Applied Chemistry and Chemical Engineering Program, Graduate School, Kogakuin University, Tokyo 192-0015, Japan; bd19003@ns.kogakuin.ac.jp; 2 Electrical Engineering and Electronics Program, Graduate School, Kogakuin University, Tokyo 192-0015, Japan; cm21051@ns.kogakuin.ac.jp (T.M.); nagai@cc.kogakuin.ac.jp (H.N.); 3 Department of Applied Physics, School of Advanced Engineering, Kogakuin University, Tokyo 192-0015, Japan

**Keywords:** photoelectrode, single-walled carbon nanotube/titania composite, conductive thin film, molecular precursor

## Abstract

A single-walled carbon nanotube/anatase (SWCNT/anatase) composite thin film with a transmittance of over 70% in the visible-light region was fabricated on a quartz glass substrate by heat treating a precursor film at 500 °C in air. The precursor film was formed by spin coating a mixed solution of the titania molecular precursor and well-dispersed SWCNTs (0.075 mass%) in ethanol. The anatase crystals and Ti^3+^ ions in the composite thin films were determined by X-ray diffraction and X-ray photoelectron spectroscopy, respectively. The effect of the heating process on the SWCNTs was analyzed using Raman spectroscopy. The composite film showed an even surface with a scratch resistance of 4H pencil hardness, as observed using field-emission scanning electron microscopy and atomic force microscopy. The electrical resistivity and optical bandgap energy of the composite thin film with a thickness of 100 nm were 6.6 × 10^−2^ Ω cm and 3.4 eV, respectively, when the SWCNT content in the composite thin film was 2.9 mass%. An anodic photocurrent density of 4.2 μA cm^−2^ was observed under ultraviolet light irradiation (16 mW cm^−2^ at 365 nm) onto the composite thin film, thus showing excellent properties as a photoelectrode without conductive substrates.

## 1. Introduction

Photoelectrodes with a large area and high activity have attracted attention as essential for photovoltaic cells, sensors, and water-splitting devices [1,2,3,4,5]. Anatase, a crystal structure of titanium dioxide (titania), is an *n*-type semiconductor that can be excited by ultraviolet (UV) light irradiation with a wavelength shorter than approximately 388 nm, corresponding to the bandgap energy. However, the high resistivity of titania (10^12^ Ω cm at 25 °C), which is related to enhanced electron–hole recombination, is a serious disadvantage, and facile photoelectrode fabrication without conductive substrates is still challenging [6,7].

Since their discovery by Iijima in 1991 [8], carbon nanotubes (CNTs) have emerged as promising nano-electronic materials because of their excellent electrical properties and thermal stability in oxidizing environments. Many researchers have reported that the presence of CNTs in a titania matrix can delay or hinder the recombination of electrons and holes. However, Chen et al. reported that it is still a great challenge to disperse CNTs uniformly inside a TiO_2_ nanoparticle matrix because of the rapid hydrolysis of the titania precursors using a conventional sol–gel method [9,10]. Despite these difficulties, Morales et al., fabricated CNT/TiO_2_ thin films using a sol–gel dip-coating method in which the CNT concentration was 4% in the sol [11]. The electrical resistivity on the order of 10^2^ and 10^5^ Ω cm during UV irradiation and in the dark condition, respectively, were recorded for the obtained composite films. However, to the best of our knowledge, there have been no reports on the lower resistivity of translucent titania composite thin films with CNTs.

The molecular precursor method (MPM) is a wet chemical process that we have developed to fabricate nanocrystalline thin films of metals and various metal oxides and phosphates [12,13,14]. The MPM uses coating solutions dissolving designed metal complex salts. These coating solutions have many practical advantages, such as excellent stability, miscibility, and coatability. Recently, we reported the fabrication of multi-walled carbon nanotube (MWCNT)/SiO_2_ conductive thin films fabricated by this method [14]. The SiO_2_ precursor solution involving Si^4+^ complex with an oxalato ligand can easily provide a precursor solution for a composite thin film in which commercially available MWCNTs are uniformly dispersed. It is generally difficult to obtain a uniform dispersion of MWCNTs with a high content in a coating solution because of their easy aggregation by strong van der Waals interactions. Thus, the MPM is a unique way to overcome these interactions between CNT molecules and to obtain a uniformly dispersed precursor for CNT composite thin films. In addition, single-walled carbon nanotubes (SWCNTs) tend to be easily damaged during the formation process, whereas SWCNT composites are suitable for making more conductive composites [15,16].

Based on our achievements in the transparent thin-film fabrication of highly sensitive titania by the MPM [17], a composite thin-film electrode comprising SWCNTs and titania was investigated. Herein, we report the facile fabrication of a translucent and highly conductive SWCNT/anatase thin film well adhered onto a quartz glass substrate by heat treating the spin-coated molecular precursor film. In contrast to the aforementioned sol–gel case, the electrical resistivity of the composite thin-film electrode was unprecedentedly low, 6.6 × 10^−^^2^ Ω cm, even when the SWCNT content in the mixed precursor solution was extremely low, 0.075 mass%. Owing to the autonomous conductivity of the composite thin-film electrode, the anodic photocurrent density could be directly measured under UV-light irradiation. In terms of the SWCNT changes during the formation process of the composite, various properties of the composite thin film as a novel conductive photoelectrode and heat-treated SWCNT films were examined and compared with those of a titania thin-film alone.

## 2. Materials and Methods

### 2.1. Materials

An ethanolic titania precursor solution (TFLEAD-Ti, Ti^4+^ = 0.5 mmol g^−^^1^) and 4A molecular sieves were procured from FUJIFILM Wako Pure Chemical Corporation, Osaka, Japan. 2-Propanol and an ethanol solution (eDIPS INK) in which 0.2 mass% of SWCNTs were dispersed were purchased from Taisei Chemical Co., Ltd., Tokyo, Japan and Meijo Nano Carbon Co., Ltd., Aichi, Japan, respectively. Deionized water was purchased from Kyoei Pharmaceutical Co., Ltd. (Chiba, Japan). Ethanol was acquired from Ueno Chemical Industries, Ltd., Tokyo, Japan, and dried on 4A molecular sieves before use. The other materials were used as received without further purification. Polished quartz glass plates were acquired from Akishima Glass Co., Ltd., Tokyo, Japan, and quartz glass substrates with dimensions of 20 × 33 × 1.5 mm^3^ were prepared. They were cleaned in 2-propanol using an ultrasonic bath to remove contaminants from the surfaces, followed by rinsing several times with deionized water. The substrates were then dried in a drying oven at 70 °C.

### 2.2. Preparation of Titania Precursor Solution, SWCNT-Dispersed Solution, and SWCNT/Titania Precursor Solution

A titania precursor solution (**S_Titania_**) was prepared by diluting 9.0 g of the molecular precursor solution (TFLEAD-Ti) with 1.0 g of ethanol. The solution was stirred using a magnetic stirrer for 1 h at room temperature. The concentration of Ti^4+^ ions was 0.45 mmol g^−1^. The SWCNT-dispersed solution (**S_CNT_**) was prepared by diluting 1.0 g of eDIPS INK with 3.0 g of ethanol, followed by stirring with a magnetic stirrer for 1 h at room temperature. The SWCNT/titania precursor solution (**S_COMP_**) was prepared by mixing 10.0 g of TFLEAD-Ti and 6.0 g of eDIPS INK. The mixed solution was stirred using a magnetic stirrer for 1 h at room temperature. The concentration of Ti^4+^ ion in **S_COMP_** was 0.31 mmol g^−1^ and that of SWCNT was 0.075 mass%.

### 2.3. Fabrication of Thin-Film Electrodes and Heat-Treated SWCNT Films

**Scheme 1:**
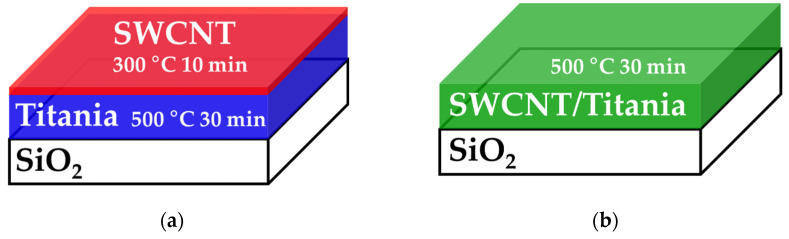
The schematic illustration of (**a**) **F_Titania_** and (**b**) **F_COMP_**.

Two types of thin-film electrodes were fabricated using the prepared precursor solutions. In all cases, a spin-coating method at room temperature was applied to spread each dropped solution (0.2 mL) in two steps, first at 500 rpm for 5 s, then at 2000 rpm for 30 s. The obtained precursor films were subsequently preheated in a drying oven at 70 °C for 10 min before subsequent treatment. First, **S_Titania_** was dropped using a micropipette and spin coated onto a 20 × 33 mm^2^ area of a quartz glass substrate as the first layer. The precursor film was then heat treated at 500 °C for 30 min in air. On top of the obtained thin film, the SWCNT precursor film was applied as the second layer using **S_CNT_**, followed by the aforementioned spin-coating and preheating processes. The SWCNT precursor film was then heat treated at 300 °C for 10 min in air. The resultant layered thin-film electrode is denoted **F_Titania_**. Second, the SWCNT composite thin-film electrode (**F_COMP_**) was fabricated by utilizing **S_COMP_** and an identical procedure as the first layer of **F_Titania_**. The schematic structure of the two electrodes is illustrated in Scheme 1.

Three types of SWCNT films were additionally formed on quartz glass substrates, as follows: an SWCNT film, **F_CNT_**, was obtained by the aforementioned spin coating and preheating of **S_CNT_**. The other two films were obtained by heat treatment of **F_CNT_** at 300 °C for 10 min and at 500 °C for 30 min in air to form **F′_CNT_** and **F″_CNT_**, respectively.

### 2.4. Structural Characterization of Thin-Film Electrodes and SWCNT Films

The crystal structures of **F_Titania_** and **F_COMP_** were determined using X-ray diffraction (XRD; SmartLab, Rigaku, Tokyo, Japan) with a Cu-*Kα* radiation source at a power of 45 kV and 200 mA. Parallel beam optics at an incidence angle of 0.3° were used in the 2θ range of 10–80°, scanning at a 0.05° step width and a speed of 5° min^−1^.

The Raman spectra of five samples, **F_Titania_**, **F_COMP_**, **F_CNT_**, **F′_CNT_**, and **F″_CNT_**, were measured using a Raman microspectrometer (LaBRAM HR800, Horiba Ltd., Kyoto, Japan) with a charge-coupled device detector. A Nd:YAG laser (532 nm) was used as the excitation source with an intensity of 13 mW, and spectra in the range of 1200–1720 cm^−1^ were obtained by exposing the samples to the laser beam for 60 s. The spectra were measured in back-scattering geometry, and the spot diameter of the laser light was 1 μm. Before the sample measurement, a silicon reference with a Raman peak at 520.6 cm^−1^ was used for wavelength calibration. Raman spectra of the samples were quantitatively analyzed using Origin 2018 (OriginLab Corporation, Northampton, MA, USA). The linear baseline of the Raman spectra was first subtracted, and three peaks were deconvoluted by a nonlinear least-squares method using the Voigt function. Peak fitting converged with a χ^2^ tolerance value of 1 × 10^−^^9^.

### 2.5. Chemical Characterization of Thin-Film Electrodes

X-ray photoelectron spectroscopy (XPS) spectra of **F_Titania_** and **F_COMP_** were measured using a JPS-9030 spectrometer (JEOL Ltd., Tokyo, Japan) using non-monochromatic Al-*Kα* X-ray radiation (1486.6 eV) and a step width of 0.1 eV. The thin-film surfaces were sputtered with Ar ions at an acceleration voltage of 150 V and a current density of 2.5 mA cm^−2^ for 15 s before XPS measurement.

The XPS peaks derived from the O and Ti atoms in the thin-film electrodes were quantitatively analyzed using Origin 2018 software (OriginLab Corporation, Northampton, MA, USA). The binding energies were corrected regarding the C 1s peak at 284.6 eV after the Shirley-type baseline was calculated. The Ti 2p, O 1s, and C 1s peaks were deconvoluted by a nonlinear least-squares method using the Voigt function. Peak fitting converged with a χ^2^ tolerance value of 1 × 10^−^^9^.

The O/Ti ratio of the thin-film electrodes was evaluated from the peak areas of O 1s and the deconvoluted Ti 2p_3/2_ peaks using relative sensitivity factors obtained from SpecSurf software (JEOL Ltd., Tokyo, Japan). The ratio can be represented by the following:

(1) O/Ti ratio =peak area of O 1srelative sensitivity factor of O 1speak area of Ti 2p3/2relative sensitivity factor of Ti 2p3/2

The C/Ti ratio of the thin-film electrodes was also calculated by replacing O 1s with C 1s in an identical manner. To determine the C/Ti ratios in the deeper portions of the thin-film electrodes, XPS depth profiling was performed using Ar^+^ ion sputtering at an acceleration voltage of 400 V and a current density of 8.9 mA cm^−2^ at 30 s intervals. The atomic ratios of C, N, O, Si, and Ti in the thin-film electrodes were calculated from the relative sensitivity factors and the peak areas of C 1s, N 1s, O 1s, Si 2p, and Ti 2p_3/2_, respectively.

### 2.6. Surface Roughness, Morphology, and Pencil Hardness of Thin-Film Electrodes

The roughness and surface three-dimensional (3D) views of **F_Titania_** and **F_COMP_** were obtained by atomic force microscopy (AFM) (OLS–4500, Olympus, Tokyo, Japan) by scanning each 2 × 2 µm^2^ area. The surface morphologies of **F_Titania_**, **F_COMP_**, **F_CNT_**, **F′_CNT_**, and **F″_CNT_** were observed by field-emission scanning electron microscopy (FE-SEM) using a JSM-6701F microscope (JEOL Ltd., Tokyo, Japan) at an accelerating voltage of 5 kV. The average thickness of the SWCNT bundles of each thin film was calculated from ten randomly selected bundles.

The pencil hardness of **F_Titania_** and **F_COMP_** was evaluated according to the Japanese Industrial Standard (JIS) K5400 by a pencil scratch test using an MJ-PHT pencil hardness meter (Sato Shouji Inc., Kanagawa, Japan) with a 0.75 kg load. The surfaces of the thin-film electrodes were scratched using a pencil (UNI, Mitsubishi Pencil Co., Ltd., Tokyo, Japan) with various hardness values standardized in the hardening order from 6B to 9H.

### 2.7. Optical Property and Thickness of Thin-Film Electrodes

The transmittance spectra of **F_Titania_** and **F_COMP_** were measured in the range of 200–1100 nm with a double-beam mode; air was used as the reference for each measurement. The measurements were performed using a UV-1900i spectrophotometer (Shimadzu, Kyoto, Japan). The optical bandgap energy was determined using the Tauc plot equation, which is expressed as follows:

(2) $$E=\frac{hc}{\lambda }$$

(3) $$\alpha =\frac{1}{d}\ln\left(\frac{1}{T}\right)$$

where *E* is the photon energy, *h* is the Planck constant, *c* is the speed of light (3.0 × 10^8^ m s^−1^), *λ* is the wavelength, *T* is the transmittance, *d* is the film thickness, and *α* is the absorption coefficient.

The thicknesses of **F_Titania_** and **F_COMP_** were measured using a stylus profilometer (DEKTAK-3, Sloan, CA, USA). For sample preparation, a portion of each precursor film was removed using ethanol to expose the substrate. Both the SWCNT and titania precursor films of **F_Titania_** were individually wiped from the quartz glass substrate with ethanol. The level differences at five positions between the surfaces of the substrate and the obtained thin-film electrode were measured for each sample. The film thickness was calculated as the average value of the three positions excluding the highest and lowest positions.

### 2.8. Electrical Property of Thin-Film Electrodes

The sheet resistances of **F_Titania_** and **F_COMP_** were measured using a four-probe method involving two multimeters (VOAC7512, IWATSU ELECTRIC Co., Ltd., Tokyo, Japan) and a regulated DC power supply (Model PAB 32-1.2, Kikusui Electronics Corp., Yokohama, Japan) at 25 °C. Four gold-plated tungsten probes (FELL type, K&S) were placed at intervals of 1 mm, and an added load of 0.2 kg was applied. The sheet resistances, *R_Sh_*, of **F_Titania_**, **F_COMP_**, **F_CNT_**, **F′_CNT_**, and **F″_CNT_** were calculated using Equation (4), and the electrical resistivity, ρ, of **F_COMP_** was estimated by the resistance and film thickness using Equation (5).

(4) $$R_{Sh}=cR$$

(5) ρ=cRt

where *c*, *R*, and *t* represent the correction value (4.45), electrical resistance, and film thickness, respectively.

### 2.9. Photocurrent Density of Thin-Film Electrodes

**F_Titania_** and **F_COMP_** were used as working electrodes. For both cases, a platinum plate (20 × 20 mm^2^) and double-junction Ag/AgCl were used as the counter and reference electrodes, respectively. A light-emitting diode (LED, model: CL-1503, LED head: CL-H1-365-9-1, Asahi Spectra Co., Ltd., Tokyo, Japan) was used as the UV light source with an intensity of 16 mW cm^−2^ at a wavelength of 365 nm. All measurements were performed in a 0.1 mol L^−1^ Na_2_SO_4_ solution after bubbling Ar gas at 50 mL min^−1^ for 10 min. The photocurrent densities were measured at 10 s intervals, first in the dark condition for 30 min, followed by irradiation with UV light for 30 min, and then again in the dark. The photocurrent density was recorded using a VersaSTAT 4 galvanostat/potentiostat (Princeton Applied Research Corp., Oak Ridge, TN, USA) under natural potential. The photocurrent densities of the three samples of each electrode were measured independently. The steady-state photocurrent densities (SSPD) of **F_COMP_** and **F_Titania_**, which reached a steady state after 30 min of UV irradiation, were obtained.

## 3. Results

A schematic illustration of the two thin-film electrodes fabricated in this study is shown in Scheme 1. The precursor solution for the SWCNT/titania composite thin film, **S_COMP_**, was facilely prepared by mixing the molecular precursor solution for the titania and SWCNT dispersions, which are commercially available. Importantly, the prepared precursor solution, **S_COMP_**, is quite stable, and the translucent thin-film electrode **F_COMP_** could be reproduced even three months after preparation of the solution. The following sections describe the results obtained for these two electrodes, focusing on the changes in the SWCNTs above and inside the titania layer.

### 3.1. Surface Morphology, Film Thickness, Electrical Property, and Pencil Hardness of Thin-Film Electrodes and SWCNT Films

**Figure 1:**
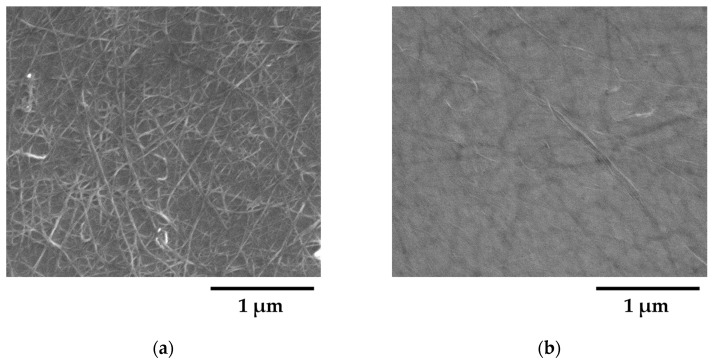
The surface morphologies of (**a**) **F_Titania_** and (**b**) **F_COMP_** observed by FE-SEM.

FE-SEM images of the thin-film electrodes are shown in Figure 1. The average diameters of the SWCNT bundles, which are clearly observed in the images of **F_Titania_** and **F_COMP,_** were 14 ± 3 and 12 ± 3 nm, respectively. The surface roughness values of **F_Titania_** and **F_COMP_** were 1.6 and 1.9 nm, respectively, as determined by the AFM 3D surface appearances (Figure S1). The maximum height difference between the highest and deepest positions deviated from the average surface of **F_Titania_** and **F_COMP_** is 20 and 27 nm, respectively. The film thickness and sheet resistance of **F_Titania_** were 110 ± 10 nm and 0.21 ± 0.01 MΩ/sq, respectively, and those of **F_COMP_** were 100 ± 10 nm and 0.44 ± 0.01 MΩ/sq, respectively. The electrical resistivity of **F_COMP_** could thus be estimated as (6.6 ± 0.7) × 10^−^^2^ Ω cm. The pencil hardness of both electrodes was 4H.

The average diameters of the SWCNT bundles of **F_CNT_**, **F′_CNT_**, and **F″_CNT_** were determined to be 17 ± 6, 15 ± 3, and 12 ± 3 nm, respectively, as shown in the FE-SEM images (Figure S2). The sheet resistances of **F_CNT_**, **F′_CNT_**, and **F″_CNT_** were 0.22 ± 0.01, 0.44 ± 0.01, and 0.61 ± 0.01 MΩ/sq, respectively.

### 3.2. XRD Patterns of Thin-Film Electrodes

**Figure 2:**
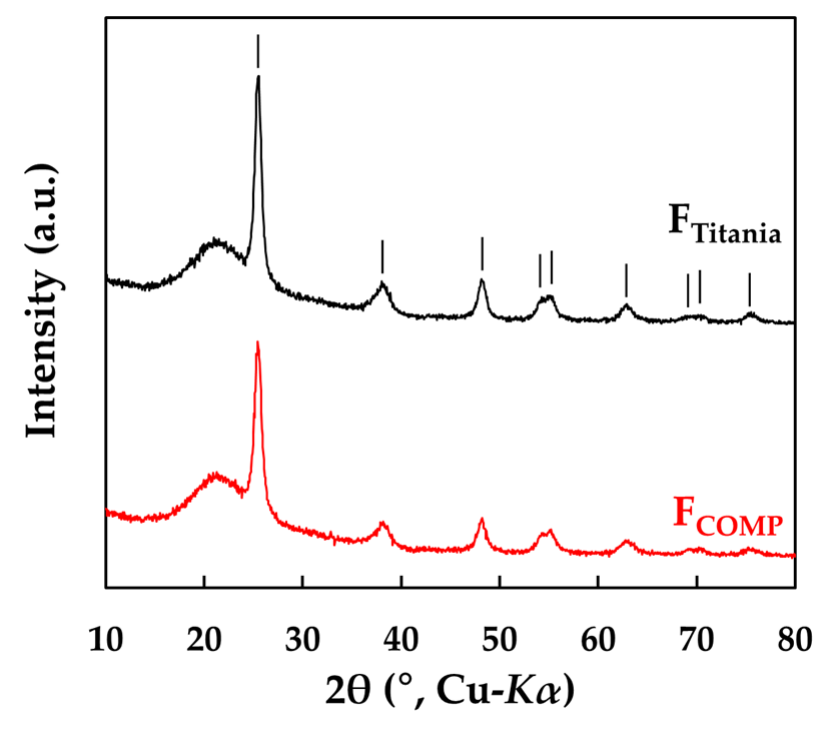
XRD patterns of **F_Titania_** and **F_COMP_**. The peak positions of anatase are specified by the indicator |.

The XRD patterns of **F_Titania_** and **F_COMP_** are shown in Figure 2. Nine peaks at 2θ = 25.5, 38.1, 48.3, 54.2, 55.4, 62.7, 69.3, 70.6, and 75.6° are assignable to the (101), (004), (200), (105), (211), (204), (116), (220), and (215) phases of anatase [ICDD 01-070-6826], respectively, in both patterns.

### 3.3. Raman Spectra of Thin-Film Electrodes, and SWCNT Films before and after Heat Treatment

**Figure 3:**
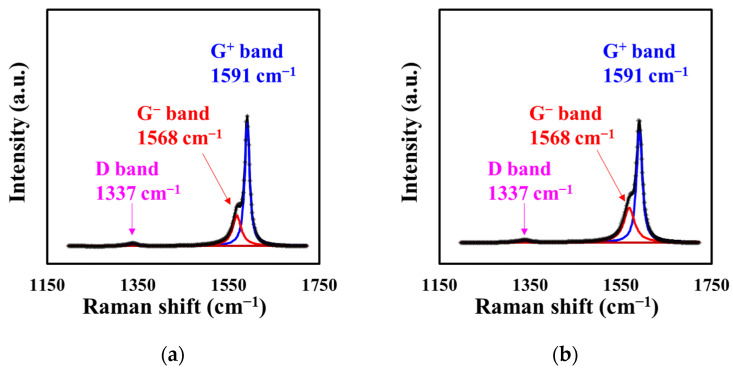
The Raman spectra of (**a**) **F_Titania_** and (**b**) **F_COMP_**. The dotted line indicates the original Raman spectra.

The Raman spectra of **F_Titania_** and **F_COMP_** are shown in Figure 3. The Raman spectra of both electrodes show three characteristic peaks at 1337, 1568, and 1591 cm^−1^ derived from the D, G^−^, and G^+^ bands of the SWCNTs, respectively [18,19]. The (G^−^ and G^+^)/D ratios of **F_Titania_** and **F_COMP_** were 46 and 47, respectively, which were calculated from the peak areas of the three bands. The (G^−^ and G^+^)/D ratios of **F_CNT_**, **F′_CNT_**, and **F″_CNT_** were also obtained as 61, 59, and 38, respectively, from the Raman peaks of the D band at 1336, 1344, and 1343 cm^−1^, the G^−^ band at 1565, 1575, and 1572 cm^−1^, and the G^+^ band at 1587, 1596, and 1594 cm^−1^, respectively (Figure S3).

### 3.4. XPS Depth Profile and XPS Spectra of Thin-Film Electrodes

**Figure 4:**
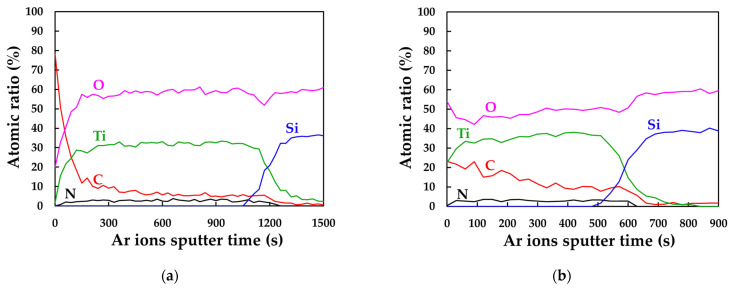
XPS depth profiles of (**a**) **F_Titania_** and (**b**) **F_COMP_**.

Figure 4 shows the XPS depth profiles of **F_Titania_** and **F_COMP_**. Both electrodes were formed of Ti and O, with small and trace amounts of C and N atoms, respectively. In the case of **F_Titania_**, extremely high concentrations of carbon atoms were observed in the range of 0–200 s of Ar^+^ ion sputter time as compared to **F_COMP_**. The calculated C/Ti ratios of **F_Titania_** and **F_COMP_** in the range of Ar^+^ ion sputter times where the ratios can be considered constant (300–990 s for **F_Titania_**; 300–480 s for **F_COMP_**) were 0.19 and 0.27, respectively.

**Figure 5:**
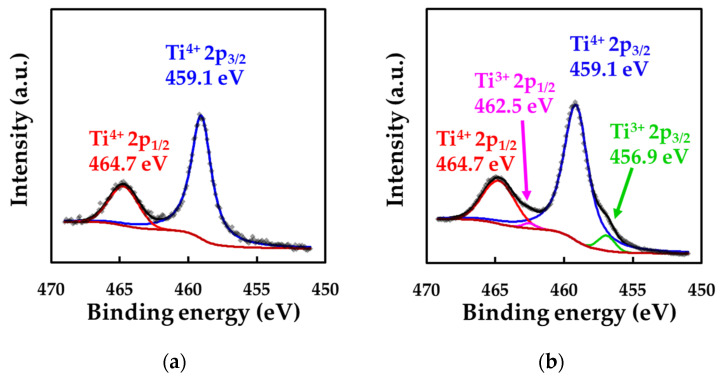
Ti 2p XPS spectra of (**a**) **F_Titania_** and (**b**) **F_COMP_** after Ar^+^ ion beam bombardment at a voltage of 150 V for 15 s. The dotted line indicates the original XPS data. The solid brown line indicates the Shirley-type baseline. The colored curves indicate theoretically fitted curves by assuming the Voigt distribution.

Figure 5 shows the XPS spectra of **F_Titania_** and **F_COMP_** in the range of 451–469 eV after Ar^+^ ion sputtering at an acceleration voltage of 150 V for 15 s, along with the deconvoluted peaks. The peaks at 459.1 and 464.7 eV for both electrodes correspond to Ti 2p_3/2_ and Ti 2p_1/2_, respectively, of Ti^4+^. In the case of **F_COMP_**, the deconvoluted peaks at 456.9 and 462.5 correspond to Ti 2p_3/2_ and Ti 2p_1/2_, respectively, of Ti^3+^ [20,21].

The deconvoluted XPS peaks corresponding to the binding energies of C 1s and O 1s orbitals were obtained for **F_Titania_** and **F_COMP_** (Figure S4). Peaks of the C 1s orbital at 286.3 and 284.6 eV, assignable to the C–O and C–C bonds, respectively, were observed for both electrodes. The O 1s spectra for **F_Titania_** and **F_COMP_** showed main peaks assignable to the O–Ti bond at 530.5 and 530.7 eV, respectively, and a shoulder for both at 532.7 eV, which is attributed to the O–C bond. **F_COMP_** showed a peak at 532.1 eV, corresponding to the O–H bond. The calculated O/Ti ratios from the peak areas of O 1s and Ti 2p_3/2_ for **F_Titania_** and **F_COMP_** were 2.0 and 1.8, respectively.

### 3.5. Transmittance Spectra and Photocurrent Density of Thin-Film Electrodes

**Figure 6:**
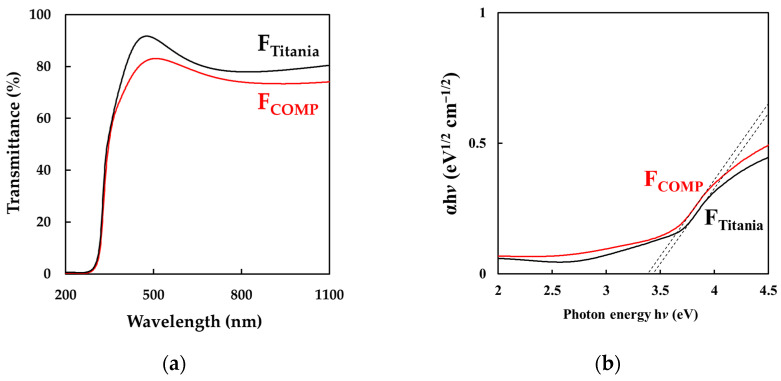
(**a**) Transmittance spectra and (**b**) Tauc plots of **F_Titania_** and **F_COMP_**.

Figure 6 shows the transmittance spectra and Tauc plots of the thin-film electrodes. The transmittance of **F_COMP_** is more than 70% in the visible-light region and 3–10% lower than that of **F_Titania_**. The optical bandgap energy of the thin-film electrodes was calculated using the Tauc plots, assuming anatase as an indirect transition semiconductor. The optical bandgap energy of both electrodes was 3.4 eV.

**Figure 7:**
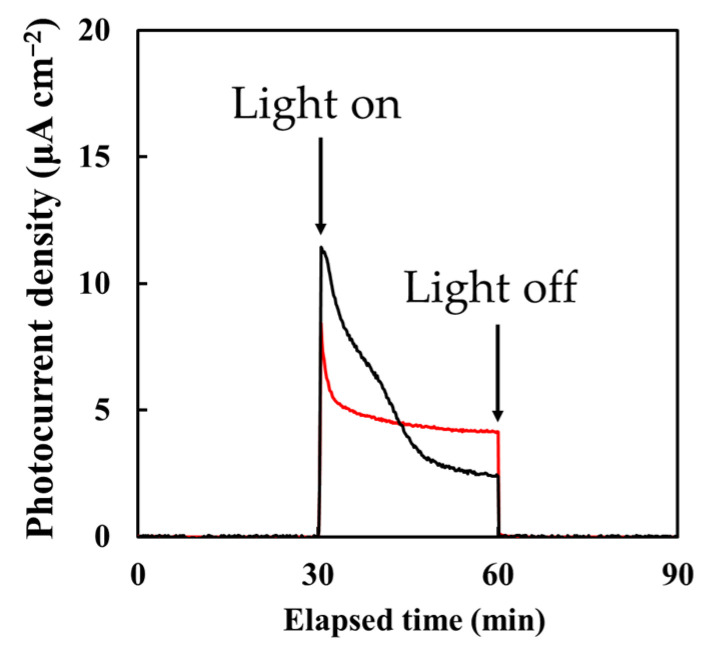
Results of the photocurrent density measurements under dark and UV irradiation of **F_Titania_** and **F_COMP_**.

The **F_Titania_** and **F_COMP_** thin-film electrodes produced anodic photocurrents at natural potential under UV-light irradiation (Figure 7). The SSPD value for **F_COMP_** of 4.2 ± 0.1 μA cm^−2^ was larger than that of **F_Titania_**, 2.5 ± 0.1 μA cm^−2^.

## 4. Discussion

### 4.1. Preparation of SWCNT/Anatase Precursor Solution and Composite Thin-Film Electrode

The SWCNT/titania precursor solution, **S_COMP_**, remained a stable SWCNT-dispersed solution for more than several months under ambient conditions. It is generally difficult to disperse CNTs uniformly in composite precursor solutions using the sol–gel method because they tend to aggregate owing to the intermolecular attractive forces, that is, van der Waals forces, between CNT molecules; reducing the surface energy and van der Waals forces can improve the dispersibility of CNT molecules [22,23]. In addition, the van der Waals interaction energy between two single molecules is very small, but that between two particles consisting of many molecules is large [24]. Molecular precursor solutions containing stable metal complex ions do not form colloidal particles through the normal hydrolysis that occurs in the presence of water [25]. Therefore, the interaction energy between CNT molecules dispersed in the molecular precursor solution is rather small compared to that in a colloidal solution, resulting in a practically stable and uniformly dispersed solution with high miscibility with CNTs.

Morales et al., reported the fabrication of a CNT/TiO_2_ thin film with a thickness and electrical resistivity of 180 nm and 2.5 × 10^5^ Ω cm, respectively, by a typical sol–gel method [11]. The dip-coated film on a quartz glass using 4 mass% CNTs in sol was heat treated at 600 °C for 1 h in air. However, the electrical resistivity of the current **F_COMP_** with a thickness of 100 nm was 10^7^ times lower than that of the sol–gel thin-film composite, even though the concentration of SWCNTs in the present composite thin films was lower at 2.9 mass%. This significant difference in the electrical resistivity suggests that the dispersion level of the embedded SWCNTs in the composite is quite different between the two methods. The volumetric fraction of SWCNTs in **F_COMP_** was calculated as 8.3% on the assumption that the densities of SWCNTs and anatase are 1.3 and 3.9 g cm^−3^, respectively [26,27]. Compared with a conventional sol–gel solution, the molecular precursor solution for titania thin-film fabrication has practical advantages toward preparing a composite precursor solution with well-dispersed CNTs, in addition to its stability and applicability to the spin-coating process.

### 4.2. Raman and XPS Spectra of Thin-Film Electrodes

The Raman peaks of **F_Titania_** and **F_COMP_** in the range of 1200–1720 cm^−1^ were successfully deconvoluted into three characteristic peaks attributable to the phononic mode of the SWCNTs (Figure 3). In both electrodes, the heat-treatment temperature to which the SWCNTs were exposed and the contact opportunity between the SWCNTs and anatase crystals significantly depended on whether the SWCNTs were above or inside the anatase layer (Scheme 1). However, the Raman spectra of both electrodes showed no significant difference in the peak positions, indicating same-level interaction between the SWCNTs and anatase.

The (G^−^ and G^+^)/D ratios of **F_Titania_** and **F_COMP_** were almost identical and approximately 20% smaller than those of **F_CNT_** and **F′_CNT_**. This indicates that the identical-level oxidation of the graphite sites in the SWCNTs occurred during heat treatment at 300 °C above the anatase layer and at 500 °C inside the anatase matrix. However, the (G^−^ and G^+^)/D ratio of **F_COMP_** was approximately 20% larger than that of **F″_CNT_**, which existed alone on a quartz glass substrate and was heat treated under the same conditions as **F_COMP_**. These results suggest that the anatase matrix effectively prevented the graphite sites of SWCNTs from experiencing thermal damage at 500 °C.

The XPS depth profiles of both **F_Titania_** and **F_COMP_** showed the presence of carbon atoms throughout the coatings, regardless of the depth (Figure 4). The C/Ti ratio of **F_COMP_** was 1.4 times that of **F_Titania_** in the portions where the concentration of carbon atoms was almost constant. This result suggests that the excess carbon atoms not contained inside **F_Titania_** are owing to the embedded SWCNTs, which were distributed evenly in the depth direction of **F_COMP_**.

The Ti 2p XPS spectra showed the coexistence of Ti^3+^ and Ti^4+^ ions in **F_COMP_** in contrast to **F_Titania_**, indicating the incorporation of O deficiency only in **F_COMP_** (Figure 5). In our previous study, an O-deficient anatase thin film containing Ti^3+^ ions was obtained by the MPM through post-annealing in air of a precursor film that included the Ti complex of ethylenediamine-N,N,N′,N′-tetraacetic acid (EDTA) and heat treatment in Ar gas flow at 500 °C [17]. In the case of the sol–gel method, a higher heat treatment at 600 °C in air was essential to crystallize titania in the composite with CNTs [11]. In this study, Ar gas was not used, and a SWCNT/O-deficient anatase thin film with Ti^3+^ ions could be formed at 500 °C, even in air. Thus, the solution process developed in this study has the advantage of crystallization of semiconducting anatase in air with coexisting Ti^3+^ and conductive SWCNTs.

### 4.3. Optical Bandgap and Photocurrent Density of SWCNT/Anatase Thin-Film Electrodes

The optical bandgap energies of **F_Titania_** and **F_COMP_** were higher than that of single-crystal anatase (3.2 eV) [28]. Generally, large bandgap energies are attributed to the stress between the thin films and the substrates [29]. The bandgap energy of **F_COMP_** was identical to that of **F_Titania_**, as shown in Figure 6b. Effective photocurrent densities were observed for both electrodes under UV-light irradiation. It is well known that when anatase particles absorb light with an energy higher than the bandgap energy, the photoexcited electrons transfer from the valence band (VB) to the conduction band; positively charged holes are simultaneously produced in the VB. Most of these carriers yielded inside ordinary particles recombine immediately before the photocurrent flows to an external circuit. However, when anatase chemically bonds to SWCNTs, photoexcited electrons are more likely to be trapped in SWCNT molecules, reducing the recombination opportunities and extending their lifetime compared to those in ordinary particles. Therefore, CNTs used in previously fabricated photoelectrodes only assist in electrically connecting the semiconductor film to a transparent conductive glass substrate (TCO) such as FTO [30]. In this study, **F_Titania_** used an SWCNT film instead of TCO, but the photocurrent density of **F_COMP_** was clearly larger than that of **F_Titania_**. A translucent and TCO-free SWCNT/anatase composite thin-film electrode with sufficient conductivity to enable photocurrent measurements without a current collector was thus obtained.

The ordinary photocurrent of titania demonstrated a rapid and sharp increase upon UV-light irradiation, and it decreased to a constant value after a certain elapsed time [31]. The results of photocurrent density measurements of **F_Titania_** and **F_COMP_** under natural potential showed the characteristic time dependence of the *n*-type semiconductor under UV-light irradiation (Figure 7). Importantly, the SSPD of **F_COMP_** was 70% higher than that of **F_Titania_**. Thus, the SWCNT/anatase composite thin-film electrode **F_COMP_** was clearly superior as a photoelectrode, although the SWCNT film on the anatase layer of **F_Titania_** can play an effective role in extracting photocurrent from an anatase thin film such as TCO.

## 5. Conclusions

An SWCNT/titania precursor solution in an ethanol solvent was facilely prepared by mixing an SWCNT-dispersed solution and a molecular precursor solution to produce a titania thin film. The stable solution was applied to a typical spin-coating process to obtain a composite precursor film on a quartz glass substrate. Subsequent heat treatment of the precursor film, which included 2.9 mass% SWCNTs, at 500 °C in air for 30 min was effective for obtaining a translucent SWCNT/titania composite thin film with an unprecedentedly low resistivity of 6.6 × 10^−^^2^ Ω cm. The XRD pattern of the composite thin film showed that the titania precursor crystallized into anatase, similar to a CNT-free precursor film subjected to the same heat treatment. Importantly, an anodic photocurrent density of 4.2 μA cm^−2^ was observed under UV-light irradiation (16 mW cm^−2^ at 365 nm) onto the composite thin film, a value that was 70% higher than that of an anatase film with the same film thickness. This suggests that the interaction between anatase and well-dispersed SWCNTs effectively decreased the recombination of photoinduced electron–hole pairs and/or enhanced the charge separation by UV-light irradiation. The XPS analyses clarified that the anatase in the composite thin film included a certain amount of Ti^3+^ ions and O deficiency as TiO_1.8_, although only O-sufficient anatase TiO_2_ was formed from the identical titania precursor alone. It was also revealed by analysis of the Raman spectra that the anatase crystals in the composite thin film prevented oxidation of the graphite sites in the embedded SWCNTs.

The SWCNT/anatase composite thin film with 1.9 nm roughness, as determined by AFM observation, had a pencil scratch hardness of 4H. The FE-SEM image of the composite thin film showed an even surface with no cracks or pinholes; it also showed SWCNT bundles 12 nm in diameter, which is smaller than those produced using lower-temperature heating or no heat treatment. The transmittance in the visible-light region and the optical bandgap energy of the SWCNT/anatase composite thin film with a thickness of 100 nm was higher than 70% and 3.4 eV, respectively.

As a result, the molecular precursor method was useful for obtaining an autonomously conductive photoelectrode, even though the SWCNT concentration in the mixed precursor solution was only 0.075 mass%. In contrast to colloids, which cannot prevent CNT aggregation, molecular precursors dissolve in solvents at the molecular level and facilitate the dispersion of CNTs. The present SWCNT/anatase composite thin film is promising as an autonomous photoelectrode that is translucent and does not require direct contact with any conductive glass substrate. We are interested in applying this photoelectrode to the assembly of a novel photovoltaic device.

## Data Availability

The data presented in this study are available on request from the corresponding author.

## Supplementary Materials

The following are available online at https://www.mdpi.com/article/10.3390/nano11123352/s1, Figure S1: Atomic force microscopy (AFM) 3D surface appearances of (a) **F_Titania_** and (b) **F_COMP_**; Figure S2: Surface morphologies of (a) **F_CNT_**, (b) **F′_CNT_**, and (c) **F″_CNT_** observed by FE-SEM; Figure S3: The Raman spectra of (a) **F_CNT_**, (b) **F′_CNT_**, and (c) **F″_CNT_**. The dotted line indicates the original Raman spectra; Figure S4: XPS spectra of (a,c) C 1s and (b,d) O 1s of **F_Titania_** and **F_COMP_**, respectively, after Ar^+^ ion beam bombardment at a voltage of 150 V for 15 s. The dotted line represents the original XPS data. The solid brown line indicates the Shirley baseline. The colored curves indicate the theoretically fitted curves by assuming a Voigt distribution.
