# Supplementary material for: Facile Fabrication of Single-Walled Carbon Nanotube/Anatase Composite Thin Film on Quartz Glass Substrate for Translucent Conductive Photoelectrode
Source: Nanomaterials (Basel). 2021 Dec 10;11(12):3352. doi: 10.3390/nano11123352 (PMC8704124; doi:10.3390/nano11123352)
Supplement: Supplementary file 1 [file nanomaterials-11-03352-s001.zip › nanomaterials-1494249-supplementary.pdf]

nanomaterials

Supplementary Materials

Facile Fabrication of Single-Walled Carbon Nanotube/Anatase Composite Thin Film on Quartz Glass Substrate for Translucent Conductive Photoelectrode

Yutaka Suwazono [1], Takuro Murayoshi [2], Hiroki Nagai [2,3] and Mitsunobu Sato [1,2,3,*]

1 Applied Chemistry and Chemical Engineering Program, Graduate School, Kogakuin University, Tokyo 192-0015, Japan; bd19003@ns.kogakuin.ac.jp
2 Electrical Engineering and Electronics Program, Graduate School, Kogakuin University, Tokyo 192-0015, Japan; cm21051@ns.kogakuin.ac.jp (T.M.); nagai@cc.kogakuin.ac.jp (H.N.)
3 Department of Applied Physics, School of Advanced Engineering, Kogakuin University, Tokyo 192-0015, Japan
* Correspondence: lccsato@cc.kogakuin.ac.jp; Tel.: +81-426-731-492

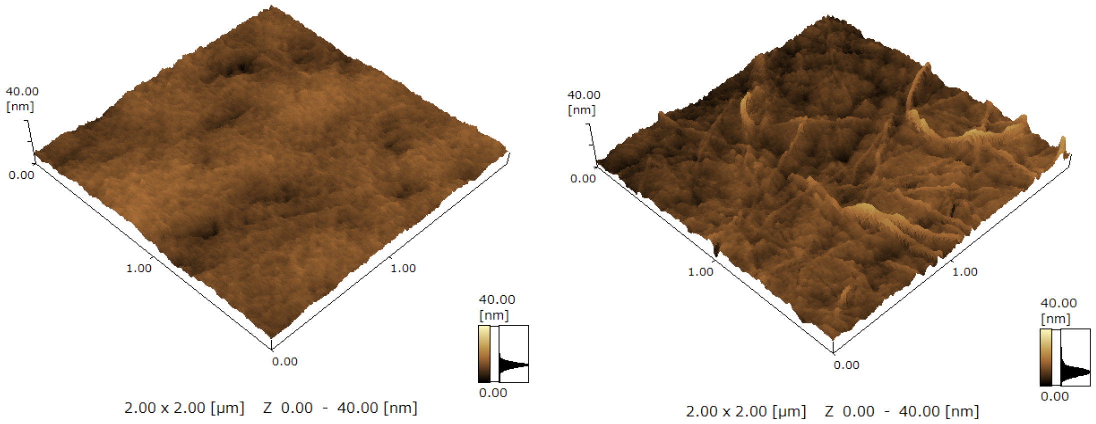

(**a**) (**b**)

Figure S1. Atomic force microscopy (AFM) 3D surface appearances of (**a**) $F_{Titania}$ and (**b**) F_{COMP}.

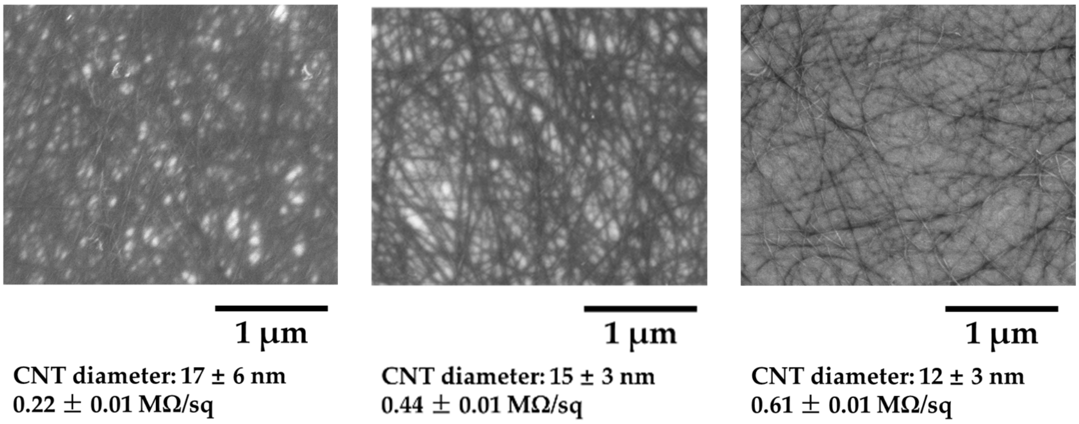

(**a**) (**b**) (**c**)

Figure S2. Surface morphologies of (**a**) F_{CNT}, (**b**) F'_{CNT}, and (**c**) F''_{CNT} observed by FE-SEM.

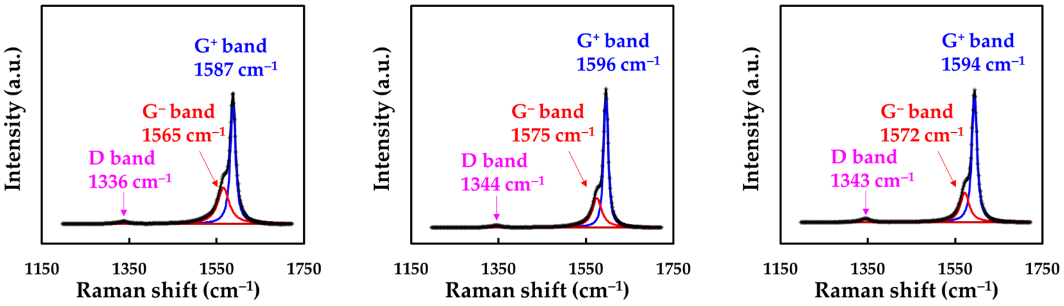

(**a**) (**b**) (**c**)

Figure S3. The Raman spectra of (**a**) $\mathbf{F_{CNT}}$, (**b**) $\mathbf{F'_{CNT}}$, and (**c**) $\mathbf{F''_{CNT}}$. The dotted line indicates the original Raman spectra.

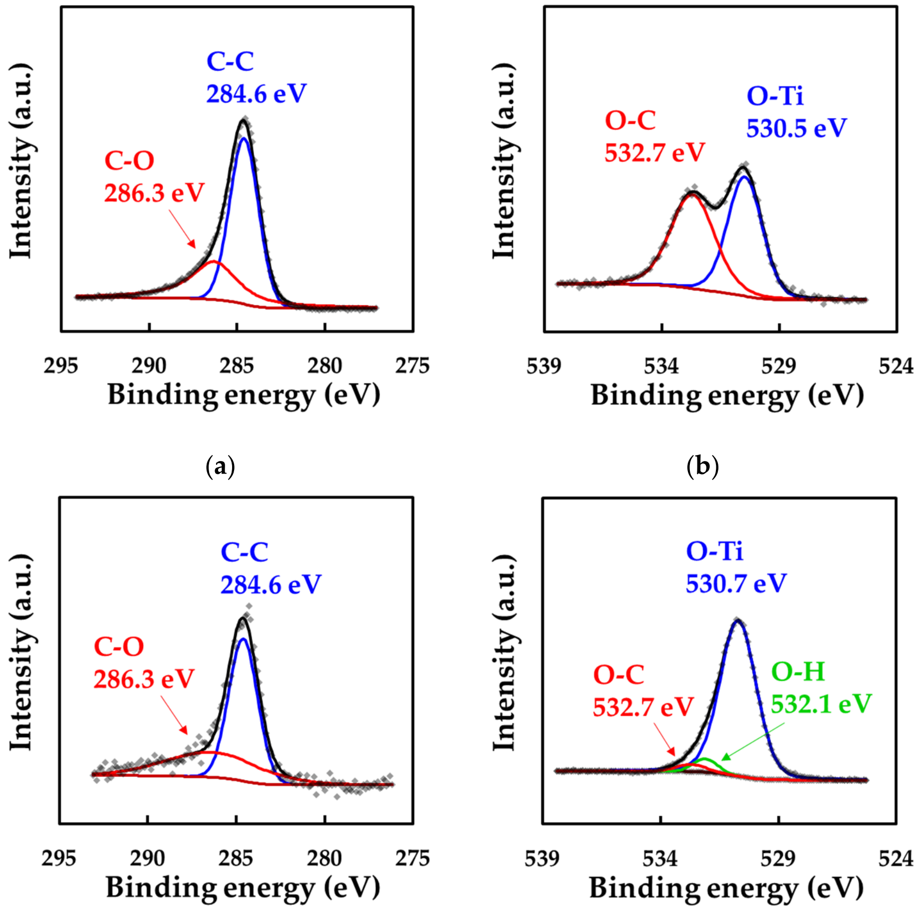

(**c**) (**d**)

Figure S4. C 1s and O 1s XPS spectra of (**a**,**b**) $\mathbf{F_{Titania}}$ and (**c**,**d**) $\mathbf{F_{COMP}}$, respectively, after Ar^{+} ion beam bombardment at a voltage of 150 V for 15 s. The dotted line represents the original XPS data. The solid brown line indicates the Shirley baseline. The colored curves indicate the theoretically fitted curves by assuming a Voigt distribution.
